# Molecular pathology of phyllodes tumours of the breast—much more than *MED12*

**DOI:** 10.1111/his.70098

**Published:** 2026-01-29

**Authors:** Jia‐Min B Pang, Kylie L Gorringe, Puay Hoon Tan, Stephen B Fox

**Affiliations:** ^1^ Peter MacCallum Cancer Centre Melbourne Victoria Australia; ^2^ Sir Peter MacCallum Department of Oncology Melbourne Victoria Australia; ^3^ Luma Medical Centre, KK Women's and Children's Hospital and Yong Loo Lin School of Medicine National University of Singapore Singapore Singapore; ^4^ Collaborative Centre for Cancer Genomic Medicine The University of Melbourne Melbourne Victoria Australia

**Keywords:** breast, molecular pathology, phyllodes tumour

## Abstract

Phyllodes tumours of the breast present challenges in their diagnosis, classification and management. Further understanding of the molecular changes underpinning these tumours may lead to more precise classification and potential treatment options. Similar to fibroadenomas, *MED12* is the most frequently mutated gene in phyllodes tumour. However, in addition, there is a spectrum of molecular alterations from benign to malignant phyllodes tumours with increasing genomic complexity, high level copy number alterations and aberrations of cancer driver genes in malignant phyllodes tumours. This review summarizes the molecular pathology of phyllodes tumours, the use of these data in developing a model of phyllodes tumour pathogenesis, and how molecular pathology might be applied to aid diagnosis and guide treatment in this rare tumour type.

## Graphical abstract

Phyllodes tumours can develop from fibroadenomas bearing *MED12* variants by the development of p*TERT* alterations (“*MED12* pathway”) or de novo (“*MED12* independent pathway”). Grade progression is associated with increasing genetic complexity including cancer driver gene aberrations. Molecular alterations can be useful in assigning grade and distinguishing phyllodes tumours from histological mimics.

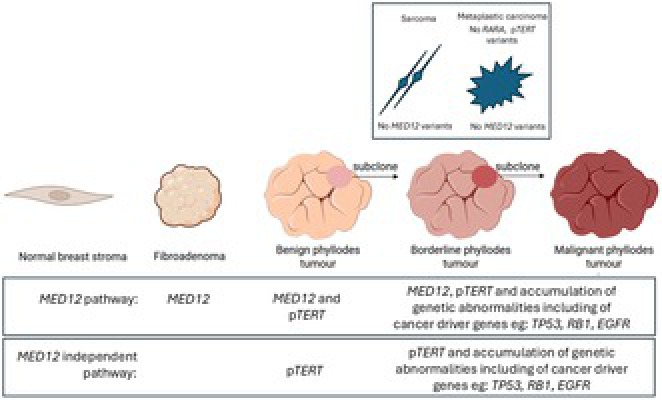

**Abbreviations**
FFPE: formalin fixed, paraffin embedded
ROR: risk of recurrence

## Introduction

Phyllodes tumours are relatively rare tumours which form part of the spectrum of fibroepithelial tumours of the breast and are graded as benign, borderline and malignant phyllodes tumours based on several histological features.^1^, ^2^ In Australia, they account for 0.2% of all breast cancers with only ~32 malignant cases per year diagnosed in 2019, equating to 0.2–0.3 per 100,000.^3^ This is similar to the incidence reported in England (2 per million women per year),^4^ USA (average annual age‐adjusted incidence 2.1 per 1 million women)^5^ and in the Netherlands (0.3 per 100,000 person years).^6^ In contrast, phyllodes tumour accounted for 6.92% of breast cancers in a Singaporean institutional study.^7^

Distinction of a phyllodes tumour from its mimics and grading of phyllodes tumour are essential for appropriate patient management.^8^ Unfortunately, this is also a recognized challenge amongst pathologists,^9^, ^10^ especially on core biopsy specimens, due to tumour heterogeneity, overlapping histological criteria, inter‐observer variation in interpretation of these criteria, and in most centres, low case volumes of phyllodes tumours. Therefore, consistent histological classification of phyllodes tumours is difficult in practice and may not strictly correlate with clinical outcome.^9^, ^10^, ^11^, ^12^

Over the last decade, identification of recurrent molecular alterations in fibroepithelial tumours has allowed greater understanding of the pathogenesis of fibroepithelial lesions and provided additional evidence for grading, risk stratification, and in differentiating phyllodes tumour from other entities including fibroadenoma and metaplastic carcinoma, and presented the possibility of targetable molecular alterations. This review aims to provide an overview of the molecular alterations in phyllodes tumours and their potential clinical utility.

## Molecular Alterations in Phyllodes Tumours

### Germline Molecular Alterations

Of the tumours that occur in patients with germline *TP53* mutations (Li‐Fraumeni syndrome), malignant phyllodes tumours have the greatest incidence relative to the general population with 78x relative frequency.^13^ Other reported pathogenic or likely pathogenic germline variants in patients with phyllodes tumours include mutations of *BRCA1, RB1, CHEK2, APC, ATM, HOXB13, MITF, RAD 51D, BARD1, MSH6, POT1, RAD51C, SDHA, MUTYH, BLM, WRN, NTHL1, POLE*, and *RECQL4*.^14^, ^15^ In a cohort of 234 women with phyllodes tumours, pathogenic or likely pathogenic germline variants occurred in approximately 14% of patients, with just of a half of these being mutations associated with an autosomal dominant pattern of inheritance, similar to that observed in patients with breast carcinoma.^14^ The prevalence of pathogenic or likely pathogenic variants increased with increasing grade of phyllodes tumour, although this difference did not reach statistical significance.^14^ Nevertheless, one might argue that this frequency is sufficient to warrant reflex germline testing in patients with phyllodes tumours.

### Somatic Alterations

While some have reported similar molecular alterations in both the stromal and epithelial components of fibroepithelial lesions,^16^, ^17^ most studies have observed molecular alterations to be confined to the mesenchymal component of the tumour,^18^, ^19^, ^20^, ^21^, ^22^ consistent with this compartment being the dominant neoplastic component of the lesion. Molecular heterogeneity within a single phyllodes tumour has been described, with differences in copy number alterations and mutations between different areas of the same tumour,^23^ and between pre‐operative core biopsy tissue and subsequent excisions.^24^ Phyllodes tumours are microsatellite stable and have a relatively low median tumour mutational burden with reported median tumour mutational burdens ranging between 2.5 mutations/MB and 2.7 mutations/MB with 1.5% to 3.3% of cases harbouring tumour mutational burdens of at least 10 mutations/MB.^25^, ^26^, ^27^

### *MED12* Mutation in Phyllodes Tumours

*MED12* is the most frequently mutated gene in phyllodes tumours (53%) and variants have been reported in all grades of phyllodes tumour^20^, ^21^, ^22^, ^23^, ^24^, ^25^, ^26^, ^27^, ^28^, ^29^, ^30^, ^31^, ^32^, ^33^, ^34^, ^35^, ^36^, ^37^, ^38^, ^39^, ^40^, ^41^, ^42^, ^43^ as well as in fibroadenomas.^19^ More than one *MED12* mutation can exist within a single tumour^28^ and *MED12* mutations can differ between fibroepithelial lesions from an individual patient.^28^, ^44^ The product of the *MED12* gene, mediator complex subunit 12, forms part of a protein complex known as mediator coactivator complex which regulates transcription either by interacting with other transcriptional regulators such as CDK8 kinase, similar to the mechanism seen in uterine leiomyomas, or directly in the case of oestrogen receptors alpha and beta.^45^, ^46^, ^47^, ^48^, ^49^, ^50^ The majority of *MED12* mutations in phyllodes tumours affect exon 2, especially codon 44,^31^ and result in loss of function of this mediator complex^51^, ^52^, ^53^ from altered positioning of the MED12 “activation helix,” which is necessary for activating CDK8. The mutations themselves do not prevent MED12 from binding CDK8 but diminish the kinase activity by misplacing this helix.^54^

### Genes Altered in Phyllodes Tumours

The true prevalence of mutations of alterations for genes (including *MED12*) is difficult to define due to the relative rarity of phyllodes tumours limiting cohort sizes, the use of a candidate gene approach in many studies focusing on likely altered genes based on the cohort composition, and different testing methodologies with a range of sensitivities and specificities. To estimate the overall frequency of alterations of individual genes, we identified published studies from PubMed using the search term “phyllodes molecular” and raw data extracted from relevant published papers and/or supplementary material associated with the papers. As the frequency of alterations is likely to be artifically skewed in reports with small numbers, a cut‐off of at least 50 cases tested was required for a gene to be included in the gene frequencies reported below.

When phyllodes tumours are regarded as a whole, the most frequently altered genes are *MED12* (53%), *TERT* promoter region (p*TERT*, 47%), *CDKN2A/CDKN2B* (44%), *MTAP* (28%), *RARA* (20%), *FLNA* (19%), *TP53* (17%), *KMT2D* (16%), *MYC* (15%), *SETD2* (15%), *NOTCH2* (13%), *TSC2* (12%), *NF1* (12%), *ARID1B* (11%), *ZBED4* (11%), *EGFR* (11%), *SYNE1* (10%), *RB1* (10%), *DNAH11* (10%), *ATM* (9%), *USH2A* (9%), *PCLO* (9%), *NRAS* (9%), *PIK3CA* (8%), *ADAMTS18* (8%), *BRAF* (8%), *BRCA2* (7%), *NTRK* (6%), *PTEN* (6%), *ERBB3* (6%), *PDGFRB* (6%), *STK11* (6%), *KIT* (6%), *CHEK2* (5%) and *RUNX1* (5%)^20^, ^21^, ^22^, ^23^, ^24^, ^25^, ^26^, ^27^, ^28^, ^29^, ^30^, ^31^, ^32^, ^33^, ^34^, ^35^, ^36^, ^37^, ^38^, ^39^, ^40^, ^41^, ^42^, ^43^ (see supplementary data).

The gene alterations consisted of single nucleotide variants alone *(MED12, FLNA, SETD2, ARID1B, ZBED4, SYNE1, PCLO, NRAS, ADAMTS18, BRCA2, ERBB3, CHEK2, RUNX1)*, copy number alterations alone *(MTAP, MYC, KIT)*, mutations and copy number alterations *(CDKN2A/CDKN2B, RARA, KMT2D, NOTCH2, TSC2, NF1, EGFR, DNAH11, ATM, USH2A, PIK3CA, PTEN, PDGFRB)*, mutations and gene fusions *(BRAF, STK11)*, as well as genes affected by mutations, copy number alterations and fusions *(pTERT, TP53, RB1, NTRK)*.^20^, ^21^, ^22^, ^23^, ^24^, ^25^, ^26^, ^27^, ^28^, ^29^, ^30^, ^31^, ^32^, ^33^, ^34^, ^35^, ^36^, ^37^, ^38^, ^39^, ^40^, ^41^, ^42^, ^43^

A single study has suggested a higher prevalence of *NF1, KMT2D*, and *RB1* mutations in metastatic tumours compared with primary tumours, as well as molecular differences associated with metastatic site, such as the presence of p*TERT* promoter, *MED12* and *RB1* mutations in lung metastases but not at other sites, and more frequent *NF1* and *KMT2D* mutations in non‐lung metastases compared with lung metastases, although these differences did not reach statistical significance.^27^ It is notable that liposarcomatous differentiation in malignant phyllodes tumours is not associated with *MDM2* and *CDK4* amplification in contrast to well‐differentiated liposarcoma and de‐differentiated liposarcoma.^55^

### Molecular Events as the Basis of a Model of Phyllodes Tumour Pathogenesis

**Figure 1:**
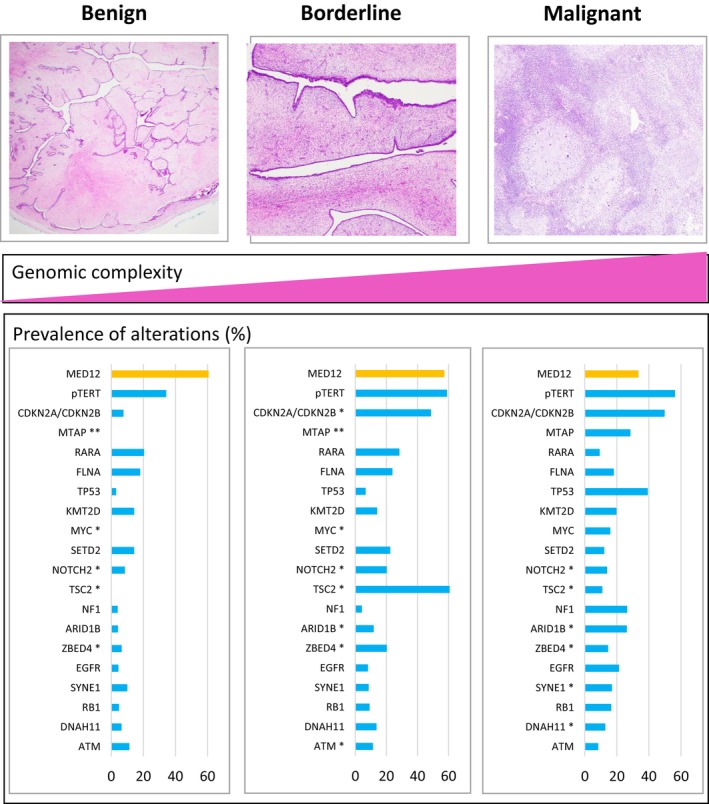
Molecular pathology of phyllodes tumours. Molecular alterations in phyllodes tumours differ with histological grade. Malignant tumours have greater complexity and increased numbers of altered genes including cancer driver genes; however, the prevalence of *MED12* mutations is lower compared with benign and borderline tumours. * indicates genes with <50 cases tested in the published literature. ** indicates genes with no cases tested in the published literature.

**Figure 2:**
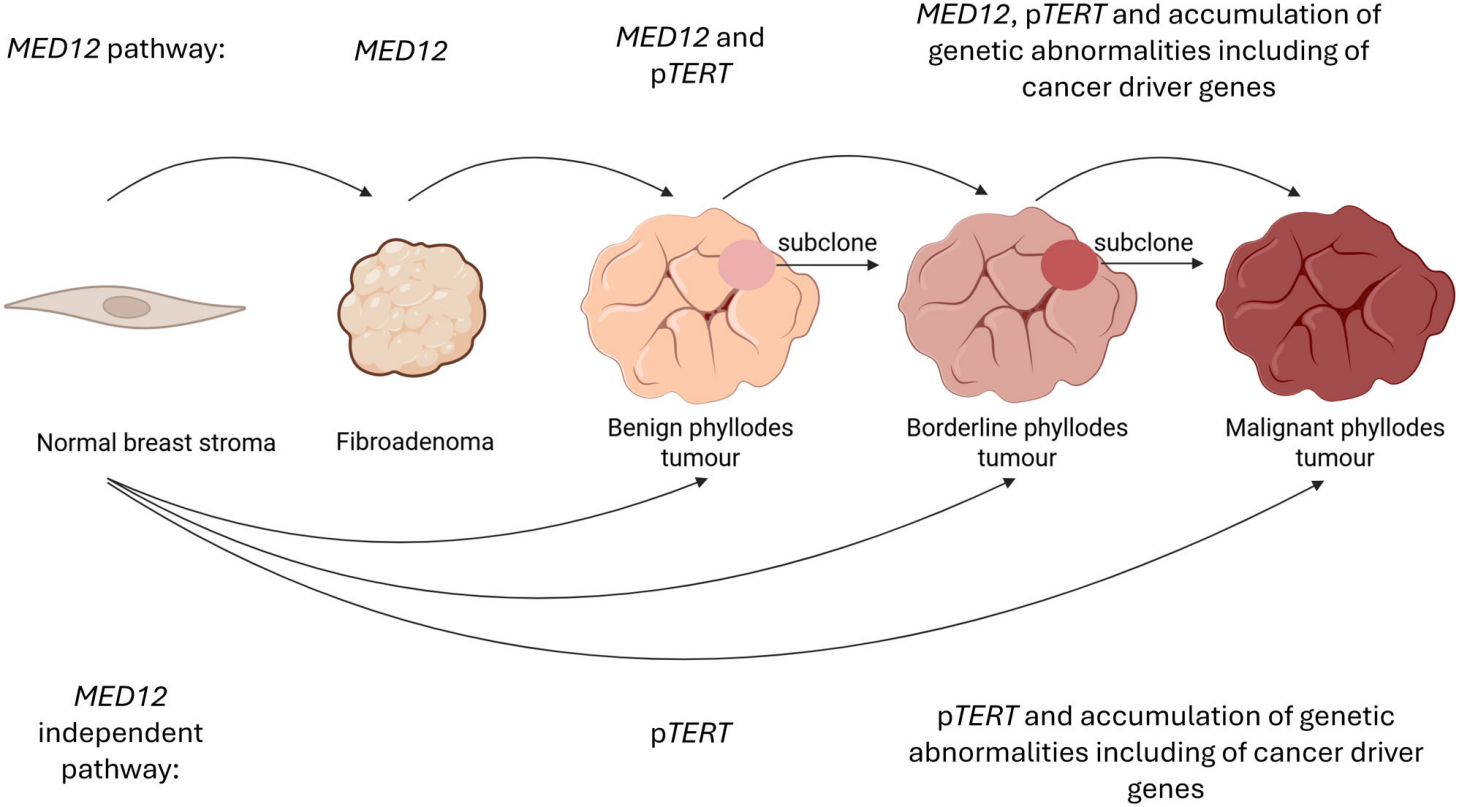
Potential pathways of phyllodes tumour pathogenesis. Phyllodes tumour can develop from *MED12‐*bearing fibroadenomas by development of p*TERT* alterations (so‐called “*MED12* pathway”). Phyllodes tumours can also develop without a pre‐existing fibroadenoma (so‐called “*MED12* independent pathway”). Progression of phyllodes tumours from benign to borderline and malignant phyllodes tumour can occur through accumulation of genetic abnormalities which may be present in a subclone of the original tumour.

Phyllodes tumours are postulated to rise through two pathways, one associated with alterations of *MED12* and the other independent of *MED12*. This hypothesis is based on the following recurrent genetic alterations observed in fibroepithelial tumours (Figures 1 and 2, supplementary data).

1. *MED12* mutations are frequently present in fibroadenomas and phyllodes tumours suggesting that *MED12* mutations are early events in the development of fibroepithelial tumours.^28^
2. The prevalence of *MED12* mutations decreases with increasing grade of phyllodes tumours with 66%, 57% and 33% variants reported in benign, borderline and malignant phyllodes tumours, respectively,^20^, ^22^, ^31^, ^33^, ^36^, ^40^.^31^, ^33^, ^36^, ^39^
3. p*TERT* alterations are exceedingly rare in fibroadenomas,^22^, ^24^, ^34^ are more prevalent in malignant (56%) and borderline phyllodes (59%) tumours compared with benign phyllodes (34%) tumours^22^, ^36^ and can be acquired in recurrent phyllodes tumours.^28^
4. *MED12* mutations are associated with p*TERT* alterations in phyllodes tumours.^34^, ^36^
5. There is greater genetic complexity with increasing grade of phyllodes tumour.^18^, ^22^, ^31^, ^32^, ^36^, ^56^
6. Alterations of cancer driver genes *TP53, RB1, EGFR, CDKN2A/CDKN2B, NF1, PTEN, NRAS, MTOR, ARID1B, PIK3CA* and *ERBB4* are rare in fibroadenoma and more prevalent in malignant/borderline phyllodes tumours compared with benign phyllodes tumours.^20^, ^22^, ^31^, ^32^, ^36^
7. *MED12* wild‐type fibroepithelial lesions show more frequent *TP53* and *PIK3CA* variants.^36^

On the basis of these observations, it is hypothesized that a proportion of phyllodes tumours arise from fibroadenomas. These phyllodes tumours harbour *MED12* mutations and progress from fibroadenomas to phyllodes tumours by the gain of p*TERT* alterations.^34^, ^35^ p*TERT* alterations are postulated to enable tumours to progress through a greater number of cell cycles leading to stromal proliferation in phyllodes tumours and increasing the likelihood of developing cancer driver mutations.^22^ In phyllodes tumours without pre‐existing *MED12* mutations, the de novo development of p*TERT* alterations and other cancer‐related genes is an alternative pathway to neoplasia.^22^, ^34^, ^35^ This model is further supported by the observation of higher frequency of *MED12* mutations in phyllodes tumours with areas morphologically resembling fibroadenomas compared with phyllodes tumours without fibroadenoma‐like areas.^35^ While the frequency of p*TERT* mutations or amplifications is similar in phyllodes tumours regardless of the presence of fibroadenoma‐like areas, *EGFR, RB1, TP53* and *NF1* alterations were more common in phyllodes tumours without fibroadenoma‐like areas, reaching statistical significance in the case of *EGFR* alterations.^35^ However, these findings have not been replicated in a subsequent study of malignant phyllodes tumours with and without fibroadenoma‐like areas,^57^ and both studies are limited by small cohort sizes (*n* = 16).

Case studies have also supported a linear genetic progression from fibroadenomas to phyllodes tumour and in grade progression of phyllodes tumours whereaccumulation of additional mutations occuron the background of shared genetic alterations.^44^, ^58^ However, it has also been demonstrated that recurrent tumours do not necessarily harbour the same genetic alterations as their primary tumours,^59^, ^60^ and it has been hypothesized that grade progression in recurrences results from subclones that are not easily identified in the primary tumour.^59^, ^60^

### Methylation Profiles

Methylation profiles of phyllodes tumours have been demonstrated to differ from those of normal breast tissue and non‐phyllodes breast tumours,^18^, ^61^ including differential methylation of genes relating to epithelial‐mesenchymal transition, KRAS signalling pathways and PRC2 targets.^61^ In a small series of fibroepithelial lesions including 41 phyllodes tumours and 17 fibroadenomas, methylation profiles derived from the Illumina Infinium MethylationEPIC BeadChip did not show a clear distinction between fibroadenomas and phyllodes tumours or between benign, borderline and malignant phyllodes tumours.^18^ However, using a much larger data set derived from the same methylation microarray and the Infinium HumanMethylation 450 BeadChip, including 33 breast tumours analysed by the authors and 817 breast tumours from publicly available databases, Meyer *et al*.^61^ identified 53 differentially methylated regions between malignant and non‐malignant phyllodes tumours. The most significantly differentially methylated region encompassed the promoter and part of the gene body of *HSD17B8*, a gene involved in oestrogen metabolism,^62^ and the degree of methylation was inversely correlated with gene expression, suggesting a role for methylation in regulating expression of this gene.

### Gene Expression Profiles

Expression patterns of 105 breast cancer‐related genes have been demonstrated to differ between different types of fibroepithelial lesions.^63^ Not surprisingly, hypoxia related genes, including *VEGFA, PNP, GAL, FLVCR2, NDRG1, FABP5* and *DDIT4*, and proliferation related genes, including *MKI67, CDC20, CCNB1, CENPF, BIRC5* and *NDC80*, were highly expressed in malignant phyllodes tumours compared with fibroadenomas and benign phyllodes tumours. Epithelial and luminal related genes were more highly expressed in fibroadenomas compared with benign phyllodes tumours, juvenile fibroadenomas, borderline phyllodes tumours and malignant phyllodes tumours in decreasing order of expression, perhaps related to the increasing stromal composition.^63^ Most malignant phyllodes tumours have similar gene expression profiles to basal‐like/claudin‐low breast carcinomas.^63^ High expression of genes involved in the negative regulation of apoptosis, negative regulation of the cell cycle, and in blood vessel, morphogenesis was also observed in malignant phyllodes tumours.^63^ Likewise, Gatalica *et al*.^38^ observed overexpression of genes involved in angiogenesis in malignant phyllodes tumours and Li *et al*.^64^ also showed malignant phyllodes tumours were distinct from other fibroepithelial lesions in their expression of genes related to hypoxia and angiogenesis, cancer‐associated intracellular signalling cascades, apoptosis regulation, epigenetic regulation, matrix remodelling and metastasis.^64^ In contrast, fibroadenomas, cellular fibroadenomas and benign phyllodes tumours had generally similar gene expression profiles, with differences in gene expression between benign and borderline phyllodes tumours in only a small number of genes.^64^

## Relevance of Molecular Alterations in Clinical Practice

### Molecular Distinction of Phyllodes Tumour from Fibroadenoma and in Grading of Phyllodes Tumours

Using a 16‐gene panel developed from genomic profiling of breast fibroepithelial lesions^20^ to interrogate a large cohort of tumours, including 258 conventional fibroadenomas, 45 cellular fibroadenomas, 322 benign phyllodes tumours, 117 borderline phyllodes tumours and 54 malignant phyllodes tumours, Md Nasir *et al*.^36^ demonstrated a higher number of mutations in phyllodes tumours compared with fibroadenomas in line with other studies,^20^, ^24^ including mutations of p*TERT, FLNA* and *SETD2*. The presence of p*TERT* mutation was identified as the most sensitive and specific marker differentiating phyllodes tumours from fibroadenomas, similar to previous studies reporting the absence^22^ or rarity^24^, ^34^ of p*TERT* alterations in fibroadenomas.

In another study using the same 16‐gene panel on core biopsies of 211 fibroepithelial lesions (167 fibroadenomas and 44 phyllodes tumours), 27 cases (12.8%) had molecular findings discordant with the original histological diagnosis. On histological review, the diagnosis of 5 cases was revised (2 cases of fibroadenoma reclassified as benign phyllodes tumour, 3 cases of phyllodes tumour reclassified as a fibroadenoma), amounting to 18.5% of the reviewed cases and 2.4% of the overall cohort.^24^

Ng *et al*.^65^ further interrogated the data obtained by Md Nasir *et al*.^36^ to investigate the feasibility of using molecular alterations to refine diagnoses in benign fibroepithelial lesions, whereby a diagnosis of benign phyllodes tumour would warrant complete excision.^8^ There were no statistically significant differences in copy number variations between conventional fibroadenomas, cellular fibroadenomas and benign phyllodes tumours. While benign phyllodes tumours differed from the combined group of conventional and cellular fibroadenomas by having a higher prevalence of *MED12*, p*TERT, RARA, FLNA, SETD2* and *RB1* alterations, the clinical utility was limited by the relatively low incidence of non‐*MED12* mutations. When histological review was undertaken due to the presence of cancer driver mutations (*TP53, RB1, NF1, PTEN, PIK3CA, EGFR, BCOR, ERBB4, MAP3K1, IGF1R*) or the presence of more than 2 mutations in non‐cancer driver genes (20% of the overall cohort), the original diagnosis was upgraded from fibroadenoma to benign phyllodes tumour or from benign phyllodes tumour to borderline phyllodes tumour in only 4.7% of the reviewed cases leading to a clinically significant change in diagnosis in only approximately 1% of the overall cohort.

Another study using a gene expression assay comprising five genes (*ABCA8*, *APOD*, *CCL19*, *FN1* and *PRAME*) developed to distinguish between fibroadenomas and phyllodes tumours on formalin fixed, paraffin embedded (FFPE) core biopsies was found to correlate with histological diagnoses in 213 of 230 cases (92.6%), with a sensitivity of 82.9% and specificity of 94.7%.^66^

Nevertheless, the absence of a tight concordance between histological diagnosis and molecular findings suggests that routine molecular testing of fibroepithelial lesions would be generally of low diagnostic yield, including the detection of p*TERT* variants and the use of surrogates such as p53 and RB1 immunohistochemistry in differentiating between fibroadenoma and benign phyllodes tumours, where p*TERT, TP53* and *RB1* variants occur in 33%, <3% and <5% of benign phyllodes tumours, respectively (supplementary data). Thus, any testing currently available may not justify the additional resources required except in select cases where there is a diagnostic dilemma, such as demonstrated in a case report where the tumour had *MED12* and p*TERT* mutations but no cancer driver alterations, resulting in a diagnosis of borderline phyllodes tumour being rendered over malignant phyllodes tumour.^67^ However, one might argue this is more of an academic exercise as it may not significantly influence patient management.

### Molecular Alterations Aid Distinction between Phyllodes Tumours and Non‐fibroepithelial Tumours

The marked stromal proliferation of phyllodes tumour can raise the differential diagnoses of metaplastic carcinoma, fibromatosis or sarcoma when the typical architecture and epithelial component of phyllodes tumour are absent or sparse. While the distinction between phyllodes tumour and soft tissue tumours may not necessarily alter treatment, metaplastic carcinoma differs from these entities in terms of chemotherapy regimens and recommended surgical margin clearances and therefore the distinction from metaplastic carcinoma is of particular importance.^8^ In this situation, demonstration of a *MED12* variant is most useful. In contrast to phyllodes tumours, *MED12* mutations are not identified in metaplastic breast carcinoma and sarcoma,^68^ and only rarely occur in fibromatosis.^39^ In addition, *RARA* and p*TERT* mutations are not seen in metaplastic carcinoma.^69^ The utility of molecular testing in cases where there is morphological and immunophenotypical overlap between phyllodes tumours, metaplastic carcinoma and breast sarcoma has been demonstrated in case reports with the presence of *MED12* and/or p*TERT* variants establishing the diagnosis of malignant phyllodes tumour.^67^, ^70^, ^71^

### Molecular Alterations as a Marker of Recurrence

Several studies have suggested that molecular alterations can be determinants of disease recurrence in phyllodes tumour in addition to traditional parameters such as tumour grade and margin status. In a small group of 19 patients with phyllodes tumours, the presence of high‐level amplifications and homozygous deletions, including in regions encompassing *MDM4, RAF1, EGFR, PDZD2* and *CDKN2A*, were only detected in tumours of patients who subsequently developed recurrent disease (local recurrence, metastasis or death) including histologically benign phyllodes tumours.^72^ The presence of *MED12* mutations has been associated with improved disease‐free survival in a cohort of 97 phyllodes tumours with a median follow up of 30 months,^33^ but this has not been replicated in other studies.^39^, ^57^ Tsang *et al*.^31^ in a cohort of 49 benign, 25 borderline and 14 malignant phyllodes tumours, reported the presence of *RARA* mutation was associated with recurrent disease but this was no longer statistically significant when cases with positive surgical margins were excluded. However, recurrence was significantly associated with the absence of epigenetic pathway alterations (defined as variants of *ARID1B, ATRX, BCOR, CHD4, CHD8, KMT2C, KMT2D, SETD2, SMARCA4* and *TET3*) regardless of surgical margin status. In a retrospective study including 33 phyllodes tumours, gene expression assessed by the PAM50 risk of recurrence (ROR) score was more predictive of clinical outcome than morphological classification of phyllodes tumours; however, the number of recurrent events was limited to 7 cases.^63^

### Molecular Alterations Identify Potential Targets for Therapy

Although MED12 is involved in oestrogen mediated pathways, the presence of *MED12* alterations is not correlated with ER protein expression.^73^ In addition, ER alpha, the well‐established biomarker indicating potential response to antioestrogen treatment, is only expressed in the epithelial component and not in the neoplastic stromal component of phyllodes tumours,^73^, ^74^, ^75^, ^76^ while ER beta is expressed in the stromal component.^73^, ^75^ The therapeutic significance of ER beta receptor expression is unknown due to uncertainties regarding its biological function in breast cancer, the presence of multiple isoforms and non‐standardized methodologies for detection.^77^, ^78^, ^79^

As systemic treatment options for advanced phyllodes tumours are limited,^80^ it is hoped that detection of molecular alterations in phyllodes tumour would allow the use of targeted therapies as in other tumour types.^81^, ^82^, ^83^ Targetable variants identified in phyllodes tumours include *EGFR* amplifications and *CDKN2A/CDKN2B* deletions,^18^, ^20^, ^23^, ^35^ variants of *AKT1, EGFR, ERBB2, ERBB3, FGFR1, FGFR2, FGFR3, BRAF, NF1, NRAS, KRAS, PIK3CA, BRCA1, BRCA2* and *PTEN*,^20^, ^23^, ^25^, ^26^, ^35^, ^84^, ^85^ and fusions involving *NTRK, BRAF* and *FGFR3*.^25^, ^27^ A very small proportion of malignant phyllodes tumours have a high tumour mutational burden which may indicate response to immunotherapy.^26^ Documented incidences of successful targeted therapy include a patient treated with larotrectinib for a pathogenic *NTRK1* fusion,^27^ complete pathological response in a lung metastasis following treatment with a personalized multi‐epitope peptide neoantigen nano‐vaccine developed based on whole exome sequencing, RNA sequencing and new antigen prediction,^86^ and a patient with a high tumour mutational burden demonstrating response to pembrolizumab.^87^ In addition, certain genetic alterations may indicate lack of response to treatment; for example, in a retrospective study, the presence of *CDKN2A* and p*TERT* alterations was associated with reduced sensitivity to chemotherapy and *CDKN2B* alterations associated with absence of response to chemotherapy, although these relationships did not reach statistical significance.^87^ Nevertheless, we have seen a clinical response to pazopanib in a patient with metastatic malignant phyllodes tumour with high VEGF expression that was likely driven by a p*TERT* variant, demonstrating its downstream effects of signalling on hypoxia and angiogenesis.^88^ Models such as patient‐derived xenografts or organoids as with other tumours might help in defining optimal therapies for patients, although limited by the known heterogeneity.^89^

## Conclusions

Over a decade of molecular research on phyllodes tumours has provided insights into their pathogenesis. While molecular testing is not currently part of routine pathological assessment, molecular profiling can be a useful adjunct in the clinically important distinction from non‐phyllodes tumours such as metaplastic carcinoma through the detection of *MED12*, p*TERT* and *RARA* variants, and to refine histological grading by identification of cancer driver genes in very selected cases. As these genes are not generally targeted in routine molecular panels for common tumours such as lung carcinomas, access to such testing may be limited in many pathology laboratories.

Genetic testing in patients with phyllodes tumour is also a valid consideration given the similar prevalence of germline mutations of autosomal dominant cancer susceptibility genes, including *TP53*, to the breast carcinoma population.^14^

Recently, less stringent criteria for the diagnosis of malignant phyllodes tumour have been proposed based on the malignant behaviour of some histologically borderline tumours,^12^ which will be incorporated into the 6^th^ Edition of the WHO Breast Tumour Classification Blue Book. It will be interesting to note the correlation of molecular alterations with revised histological grade, especially cancer driver genes alterations which are present in a proportion of tumours currently classified as borderline phyllodes tumours.

Most studies have used a candidate gene approach to detect known DNA sequence variants and potentially structural variants. Therefore, information outside of these targets, including epigenetic and expression data, is limited. Agnostic approaches analysing the whole genome/transcriptome may provide a more comprehensive picture of the molecular changes in phyllodes tumours as technologies become more accessible in the future, including mutational signatures that might shed light on the aetiology and cell of origin. Multi‐institution research collaborations will help overcome the relatively low frequency of phyllodes tumours, especially malignant phyllodes tumours, and lead to more robust data regarding the link between molecular alterations, response to targeted therapies and clinical outcomes.

## Author contributions

JMBP performed literature review, wrote and revised the manuscript. KLG wrote and reviewed the manuscript. PHT and SBF conceptualized, wrote and reviewed the manuscript. All authors approve the final version of the manuscript, figures and supplementary material for publication.

## Funding information

SBF is in receipt of a National Health and Medical Research Council grant: GNT1193630.

## Conflict of interests

The authors declare no conflicts of interest in relation to the subject matter discussed in this manuscript.

## Supporting information


**Data S1.** Prevalence of alterations by gene and phyllodes tumour grade in published studies.

## Data Availability

The authors will provide all data on request.

## References

[1] WHO Classification of Tumours Editorial Board . Breast Tumours. 5th ed. Lyon, France: International Agency for Research on Cancer (IARC), 2019.

[2] Tan BY , Acs G , Apple SK *et al*. Phyllodes tumours of the breast: a consensus review. Histopathology 2016; 68; 5–21. 26768026 10.1111/his.12876 PMC5027876

[3] Cancer Data in Australia . Welfare AIoHa, Australia 2024. 2024.

[4] Ahmed M , Collins S , Franks J *et al*. Incidence and outcome of breast sarcomas in England (2013‐2018): an analysis from the National Cancer Registration and analysis service. Eur. J. Cancer 2022; 174; 48–56. 35970036 10.1016/j.ejca.2022.06.036

[5] Bernstein L , Deapen D , Ross RK . The descriptive epidemiology of malignant cystosarcoma phyllodes tumors of the breast. Cancer 1993; 71; 3020–3024. 8387873 10.1002/1097-0142(19930515)71:10<3020::aid-cncr2820711022>3.0.co;2-g

[6] Louwman MW , Vriezen M , van Beek MW *et al*. Uncommon breast tumors in perspective: incidence, treatment and survival in The Netherlands. Int. J. Cancer 2007; 121; 127–135. 17330844 10.1002/ijc.22625

[7] Tan PH , Jayabaskar T , Chuah KL *et al*. Phyllodes tumors of the breast: the role of pathologic parameters. Am. J. Clin. Pathol. 2005; 123; 529–540. 15743740 10.1309/U6DV-BFM8-1MLJ-C1FN

[8] NCCN Clinical Practice Guidelines in Oncology (NCCN Guidelines) for Phyllodes Tumour version 4.2025. 2025. Available from: https://www.nccn.org/professionals/physician_gls/pdf/breast.pdf.

[9] Tan PH . Refining the classification of breast phyllodes tumours. Pathology 2023; 55; 437–448. 37085395 10.1016/j.pathol.2023.02.001

[10] Tan BY , Fox SB , Lakhani SR , Tan PH . Survey of recurrent diagnostic challenges in breast phyllodes tumours. Histopathology 2023; 82; 95–105. 36468287 10.1111/his.14730

[11] Turashvili G , Ding Q , Liu Y *et al*. Comprehensive clinical‐pathologic assessment of malignant phyllodes tumors: proposing refined diagnostic criteria. Am. J. Surg. Pathol. 2023; 47; 1195–1206. 37694517 10.1097/PAS.0000000000002109

[12] Tan PH , Ellis IO , Allison KH *et al*. Malignant phyllodes tumours of the breast: the case for revising WHO's ‘full house’ diagnostic criteria. Histopathology 2025; 87; 169–182. 40223225 10.1111/his.15455 PMC12232237

[13] Birch JM , Alston RD , McNally RJ *et al*. Relative frequency and morphology of cancers in carriers of germline TP53 mutations. Oncogene 2001; 20; 4621–4628. 11498785 10.1038/sj.onc.1204621

[14] Rosenberger LH , Thomas SM , Hieken TJ *et al*. Germline genetic mutations in a multi‐center cohort of 248 phyllodes tumors. Breast Cancer Res. Treat. 2025; 209; 275–282. 39269552 10.1007/s10549-024-07488-3 PMC11786992

[15] Rosenberger LH , Thomas SM , Nimbkar SN *et al*. Germline genetic mutations in a multi‐center contemporary cohort of 550 phyllodes tumors: an opportunity for expanded multi‐gene panel testing. Ann. Surg. Oncol. 2020; 27; 3633–3640. 32504368 10.1245/s10434-020-08480-z PMC9945652

[16] Waitzberg AFL , Ferreira ENE , Pinilla M *et al*. Are both distinct epithelial and stromal cells molecular analysis from phyllodes tumors versus fibroadenoma components affected in breast fibroepithelial progression? Acta Cir. Bras. 2023; 38; e386823. 38055384 10.1590/acb386823 PMC10695188

[17] Sawyer EJ , Hanby AM , Ellis P *et al*. Molecular analysis of phyllodes tumors reveals distinct changes in the epithelial and stromal components. Am. J. Pathol. 2000; 156; 1093–1098. 10702425 10.1016/S0002-9440(10)64977-2 PMC1876863

[18] Hench J , Vlajnic T , Soysal SD , Obermann EC , Frank S , Muenst S . An integrated epigenomic and genomic view on phyllodes and phyllodes‐like breast tumors. Cancers (Basel) 2022; 14; 667. 35158935 10.3390/cancers14030667 PMC8833410

[19] Lim WK , Ong CK , Tan J *et al*. Exome sequencing identifies highly recurrent MED12 somatic mutations in breast fibroadenoma. Nat. Genet. 2014; 46; 877–880. 25038752 10.1038/ng.3037

[20] Tan J , Ong CK , Lim WK *et al*. Genomic landscapes of breast fibroepithelial tumors. Nat. Genet. 2015; 47; 1341–1345. 26437033 10.1038/ng.3409

[21] Yoshida M , Sekine S , Ogawa R *et al*. Frequent MED12 mutations in phyllodes tumours of the breast. Br. J. Cancer 2015; 112; 1703–1708. 25839987 10.1038/bjc.2015.116 PMC4430713

[22] Piscuoglio S , Ng CK , Murray M *et al*. Massively parallel sequencing of phyllodes tumours of the breast reveals actionable mutations, and TERT promoter hotspot mutations and TERT gene amplification as likely drivers of progression. J. Pathol. 2016; 238; 508–518. 26832993 10.1002/path.4672 PMC4962788

[23] Liu SY , Joseph NM , Ravindranathan A *et al*. Genomic profiling of malignant phyllodes tumors reveals aberrations in FGFR1 and PI‐3 kinase/RAS signaling pathways and provides insights into intratumoral heterogeneity. Mod. Pathol. 2016; 29; 1012–1027. 27255162 10.1038/modpathol.2016.97

[24] Sim Y , Ng GXP , Ng CCY *et al*. A novel genomic panel as an adjunctive diagnostic tool for the characterization and profiling of breast fibroepithelial lesions. BMC Med. Genet. 2019; 12; 142. 10.1186/s12920-019-0588-2 PMC6813086 31647027

[25] Nozad S , Sheehan CE , Gay LM *et al*. Comprehensive genomic profiling of malignant phyllodes tumors of the breast. Breast Cancer Res. Treat. 2017; 162; 597–602. 28210881 10.1007/s10549-017-4156-1

[26] Rosenberger LH , Riedel RF , Diego EJ *et al*. Genomic landscape of malignant phyllodes tumors reveals multiple targetable opportunities. Oncologist 2024; 29; 1024–1031. 39191445 10.1093/oncolo/oyae218 PMC11630793

[27] Bansal R , Adeyelu T , Elliott A *et al*. Genomic landscape of malignant phyllodes tumors identifies subsets for targeted therapy. JCO Precis. Oncol. 2024; 8; e2400289. 39637336 10.1200/PO.24.00289 PMC11634179

[28] Garcia‐Dios DA , Levi D , Shah V *et al*. MED12, TERT promoter and RBM15 mutations in primary and recurrent phyllodes tumours. Br. J. Cancer 2018; 118; 277–284. 29315289 10.1038/bjc.2017.450 PMC5785756

[29] Tsang JYS , Hui YK , Lee MA *et al*. Association of clinicopathological features and prognosis of TERT alterations in phyllodes tumor of breast. Sci. Rep. 2018; 8; 3881. 29497099 10.1038/s41598-018-22232-w PMC5832760

[30] Kim JY , Yu JH , Nam SJ *et al*. Genetic and clinical characteristics of phyllodes tumors of the breast. Transl. Oncol. 2018; 11; 18–23. 29145046 10.1016/j.tranon.2017.10.002 PMC5684533

[31] Tsang JY , Shao Y , Poon IK *et al*. Analysis of recurrent molecular alterations in phyllodes tumour of breast: insights into prognosis and pathogenesis. Pathology 2022; 54; 678–685. 35691725 10.1016/j.pathol.2022.03.008

[32] Cani AK , Hovelson DH , McDaniel AS *et al*. Next‐gen sequencing exposes frequent MED12 mutations and actionable therapeutic targets in phyllodes tumors. Mol. Cancer Res. 2015; 13; 613–619. 25593300 10.1158/1541-7786.MCR-14-0578 PMC4936398

[33] Ng CC , Tan J , Ong CK *et al*. MED12 is frequently mutated in breast phyllodes tumours: a study of 112 cases. J. Clin. Pathol. 2015; 68; 685–691. 26018969 10.1136/jclinpath-2015-202896

[34] Yoshida M , Ogawa R , Yoshida H *et al*. TERT promoter mutations are frequent and show association with MED12 mutations in phyllodes tumors of the breast. Br. J. Cancer 2015; 113; 1244–1248. 26355235 10.1038/bjc.2015.326 PMC4647876

[35] Pareja F , Geyer FC , Kumar R *et al*. Phyllodes tumors with and without fibroadenoma‐like areas display distinct genomic features and may evolve through distinct pathways. NPJ Breast Cancer 2017; 3; 40. 29043292 10.1038/s41523-017-0042-6 PMC5638820

[36] Md Nasir ND , Ng CCY , Rajasegaran V *et al*. Genomic characterisation of breast fibroepithelial lesions in an international cohort. J. Pathol. 2019; 249; 447–460. 31411343 10.1002/path.5333

[37] Yun J , Heo W , Lee ES *et al*. An integrative approach for exploring the nature of fibroepithelial neoplasms. Br. J. Cancer 2023; 128; 626–637. 36522480 10.1038/s41416-022-02064-2 PMC9938154

[38] Gatalica Z , Vranic S , Ghazalpour A *et al*. Multiplatform molecular profiling identifies potentially targetable biomarkers in malignant phyllodes tumors of the breast. Oncotarget 2016; 7; 1707–1716. 26625196 10.18632/oncotarget.6421 PMC4811491

[39] Lae M , Gardrat S , Rondeau S *et al*. MED12 mutations in breast phyllodes tumors: evidence of temporal tumoral heterogeneity and identification of associated critical signaling pathways. Oncotarget 2016; 7; 84428–84438. 27806318 10.18632/oncotarget.12991 PMC5356671

[40] Piscuoglio S , Murray M , Fusco N *et al*. MED12 somatic mutations in fibroadenomas and phyllodes tumours of the breast. Histopathology 2015; 67; 719–729. 25855048 10.1111/his.12712 PMC4996373

[41] Yoon N , Bae GE , Kang SY *et al*. Frequency of MED12 mutations in phyllodes tumors: inverse correlation with histologic grade. Genes Chromosomes Cancer 2016; 55; 495–504. 26856273 10.1002/gcc.22351

[42] Lei T , Song Y , Shen Z *et al*. Malignant phyllodes tumors with sarcomatous components: a histopathologic and molecular study. Transl. Oncol. 2025; 53; 102318. 39922047 10.1016/j.tranon.2025.102318 PMC11849116

[43] Lien HC , Huang CS , Yang YW , Jeng YM . Mutational analysis of MED12 exon 2 in a spectrum of fibroepithelial tumours of the breast: implications for pathogenesis and histogenesis. Histopathology 2016; 68; 433–441. 26109290 10.1111/his.12764

[44] Piscuoglio S , Geyer FC , Burke KA *et al*. Massively parallel sequencing analysis of synchronous fibroepithelial lesions supports the concept of progression from fibroadenoma to phyllodes tumor. NPJ Breast Cancer 2016; 2; 16035. 28721388 10.1038/npjbcancer.2016.35 PMC5515337

[45] Ravegnini G , Marino‐Enriquez A , Slater J *et al*. MED12 mutations in leiomyosarcoma and extrauterine leiomyoma. Mod. Pathol. 2013; 26; 743–749. 23222489 10.1038/modpathol.2012.203

[46] Gonzalez CG , Akula S , Burleson M . The role of mediator subunit 12 in tumorigenesis and cancer therapeutics. Oncol. Lett. 2022; 23; 74. 35111243 10.3892/ol.2022.13194 PMC8771631

[47] Thompson CM , Koleske AJ , Chao DM , Young RA . A multisubunit complex associated with the RNA polymerase II CTD and TATA‐binding protein in yeast. Cell 1993; 73; 1361–1375. 8324825 10.1016/0092-8674(93)90362-t

[48] Knuesel MT , Meyer KD , Bernecky C , Taatjes DJ . The human CDK8 subcomplex is a molecular switch that controls mediator coactivator function. Genes Dev. 2009; 23; 439–451. 19240132 10.1101/gad.1767009 PMC2648653

[49] Galbraith MD , Donner AJ , Espinosa JM . CDK8: a positive regulator of transcription. Transcription. 2010; 1; 4–12. 21327159 10.4161/trns.1.1.12373 PMC3035184

[50] Kang YK , Guermah M , Yuan CX , Roeder RG . The TRAP/mediator coactivator complex interacts directly with estrogen receptors alpha and beta through the TRAP220 subunit and directly enhances estrogen receptor function in vitro. Proc. Natl. Acad. Sci. USA 2002; 99; 2642–2647. 11867769 10.1073/pnas.261715899 PMC122401

[51] Turunen M , Spaeth JM , Keskitalo S *et al*. Uterine leiomyoma‐linked MED12 mutations disrupt mediator‐associated CDK activity. Cell Rep. 2014; 7; 654–660. 24746821 10.1016/j.celrep.2014.03.047 PMC4041330

[52] Park MJ , Shen H , Spaeth JM *et al*. Oncogenic exon 2 mutations in mediator subunit MED12 disrupt allosteric activation of cyclin C‐CDK8/19. J. Biol. Chem. 2018; 293; 4870–4882. 29440396 10.1074/jbc.RA118.001725 PMC5880139

[53] Knuesel MT , Meyer KD , Donner AJ , Espinosa JM , Taatjes DJ . The human CDK8 subcomplex is a histone kinase that requires Med12 for activity and can function independently of mediator. Mol. Cell. Biol. 2009; 29; 650–661. 19047373 10.1128/MCB.00993-08 PMC2630685

[54] Klatt F , Leitner A , Kim IV *et al*. A precisely positioned MED12 activation helix stimulates CDK8 kinase activity. Proc. Natl. Acad. Sci. USA 2020; 117; 2894–2905. 31988137 10.1073/pnas.1917635117 PMC7022176

[55] Lyle PL , Bridge JA , Simpson JF , Cates JM , Sanders ME . Liposarcomatous differentiation in malignant phyllodes tumours is unassociated with MDM2 or CDK4 amplification. Histopathology 2016; 68; 1040–1045. 26542423 10.1111/his.12898

[56] Lae M , Vincent‐Salomon A , Savignoni A *et al*. Phyllodes tumors of the breast segregate in two groups according to genetic criteria. Mod. Pathol. 2007; 20; 435–444. 17334353 10.1038/modpathol.3800756

[57] Valenza C , Trapani D , Porta FM *et al*. The pathologic and genomic evolution of primary malignant phyllodes tumors of the breast: retrospective cohort study and case‐control genomic analysis. Oncologist 2025; 30; oyaf012. 39921370 10.1093/oncolo/oyaf012 PMC11806198

[58] Tan BY , Md Nasir ND , Chang HY *et al*. Morphologic and genetic heterogeneity in breast fibroepithelial lesions‐a comprehensive mapping study. Mod. Pathol. 2020; 33; 1732–1745. 32322022 10.1038/s41379-020-0533-0

[59] Jones AM , Mitter R , Springall R *et al*. A comprehensive genetic profile of phyllodes tumours of the breast detects important mutations, intra‐tumoral genetic heterogeneity and new genetic changes on recurrence. J. Pathol. 2008; 214; 533–544. 18288784 10.1002/path.2320

[60] Xu W , Ma W , Wang D , Zhou X , Wang K , Mu K . Integrated multi‐omics profiling reveals a clinically relevant molecular feature and potential therapeutic target on phyllodes tumors of breast. Transl. Oncol. 2024; 46; 101998. 38761630 10.1016/j.tranon.2024.101998 PMC11112002

[61] Meyer B , Stirzaker C , Ramkomuth S *et al*. Detailed DNA methylation characterisation of phyllodes tumours identifies a signature of malignancy and distinguishes phyllodes from metaplastic breast carcinoma. J. Pathol. 2024; 262; 480–494. 38300122 10.1002/path.6250

[62] Ohno S , Nishikawa K , Honda Y , Nakajin S . Expression in E. Coli and tissue distribution of the human homologue of the mouse Ke 6 gene, 17beta‐hydroxysteroid dehydrogenase type 8. Mol. Cell. Biochem. 2008; 309; 209–215. 17978863 10.1007/s11010-007-9637-9

[63] Vidal M , Peg V , Galvan P *et al*. Gene expression‐based classifications of fibroadenomas and phyllodes tumours of the breast. Mol. Oncol. 2015; 9; 1081–1090. 25687451 10.1016/j.molonc.2015.01.003 PMC5528764

[64] Li X , Vail E , Maluf H *et al*. Gene expression profiling of fibroepithelial lesions of the breast. Int. J. Mol. Sci. 2023; 24; 9041. 37240386 10.3390/ijms24109041 PMC10219050

[65] Ng CCY , Md Nasir ND , Loke BN *et al*. Genetic differences between benign phyllodes tumors and fibroadenomas revealed through targeted next generation sequencing. Mod. Pathol. 2021; 34; 1320–1332. 33727697 10.1038/s41379-021-00787-w

[66] Tan WJ , Cima I , Choudhury Y *et al*. A five‐gene reverse transcription‐PCR assay for pre‐operative classification of breast fibroepithelial lesions. Breast Cancer Res. 2016; 18; 31. 26961242 10.1186/s13058-016-0692-6 PMC4784364

[67] Koh VCY , Ng CCY , Bay BH , Teh BT , Tan PH . The utility of a targeted gene mutation panel in refining the diagnosis of breast phyllodes tumours. Pathology 2019; 51; 531–534. 31272781 10.1016/j.pathol.2019.04.005

[68] Lien HC , Huang CS , Yang YW , Jeng YM . MED12 exon 2 mutation as a highly sensitive and specific marker in distinguishing phyllodes tumours from other spindle neoplasms of the breast. APMIS 2016; 124; 356–364. 26860948 10.1111/apm.12516

[69] Ng CKY , Piscuoglio S , Geyer FC *et al*. The landscape of somatic genetic alterations in metaplastic breast carcinomas. Clin. Cancer Res. 2017; 23; 3859–3870. 28153863 10.1158/1078-0432.CCR-16-2857 PMC5511565

[70] Yeong J , Thike AA , Young Ng CC *et al*. A genetic mutation panel for differentiating malignant phyllodes tumour from metaplastic breast carcinoma. Pathology 2017; 49; 786–789. 29066183 10.1016/j.pathol.2017.07.011

[71] Schwartz CJ , Krings G , Chen YY . Malignant phyllodes tumour with lymph node metastasis: a diagnostic conundrum resolved by next generation DNA sequencing. Histopathology 2024; 84; 409–411. 37706238 10.1111/his.15046

[72] Tan WJ , Lai JC , Thike AA *et al*. Novel genetic aberrations in breast phyllodes tumours: comparison between prognostically distinct groups. Breast Cancer Res. Treat. 2014; 145; 635–645. 24831776 10.1007/s10549-014-2982-y

[73] Tan WJ , Chan JY , Thike AA *et al*. MED12 protein expression in breast fibroepithelial lesions: correlation with mutation status and oestrogen receptor expression. J. Clin. Pathol. 2016; 69; 858–865. 27056456 10.1136/jclinpath-2015-203590

[74] Singh Y , Hatano T , Uemura Y *et al*. Immunohistochemical profile of phyllodes tumors of the breast. Oncol. Rep. 1996; 3; 677–681. 21594434

[75] Sapino A , Bosco M , Cassoni P *et al*. Estrogen receptor‐beta is expressed in stromal cells of fibroadenoma and phyllodes tumors of the breast. Mod. Pathol. 2006; 19; 599–606. 16554735 10.1038/modpathol.3800574

[76] Tse GM , Lee CS , Kung FY *et al*. Hormonal receptors expression in epithelial cells of mammary phyllodes tumors correlates with pathologic grade of the tumor: a multicenter study of 143 cases. Am. J. Clin. Pathol. 2002; 118; 522–526. 12375638 10.1309/D206-DLF8-WDNC-XJ8K

[77] Zhou Y , Liu X . The role of estrogen receptor beta in breast cancer. Biomark. Res. 2020; 8; 39. 32944243 10.1186/s40364-020-00223-2 PMC7487630

[78] Monteiro FL , Stepanauskaite L , Archer A , Williams C . Estrogen receptor beta expression and role in cancers. J. Steroid Biochem. Mol. Biol. 2024; 242; 106526. 38657699 10.1016/j.jsbmb.2024.106526

[79] Bozovic A , Mandusic V , Todorovic L *et al*. Estrogen receptor Beta: the promising biomarker and potential target in metastases. Int. J. Mol. Sci. 2021; 22; 1656. 33562134 10.3390/ijms22041656 PMC7914503

[80] Palassini E , Mir O , Grignani G *et al*. Systemic treatment in advanced phyllodes tumor of the breast: a multi‐institutional European retrospective case‐series analyses. Breast Cancer Res. Treat. 2022; 192; 603–610. 35150367 10.1007/s10549-022-06524-4

[81] Jeon H , Wang S , Song J , Gill H , Cheng H . Update 2025: management of non‐small‐cell lung cancer. Lung 2025; 203; 53. 40133478 10.1007/s00408-025-00801-x PMC11937135

[82] Seth R , Agarwala SS , Messersmith H *et al*. Systemic therapy for melanoma: ASCO guideline update. J. Clin. Oncol. 2023; 41; 4794–4820. 37579248 10.1200/JCO.23.01136

[83] Meyer ML , Fitzgerald BG , Paz‐Ares L *et al*. New promises and challenges in the treatment of advanced non‐small‐cell lung cancer. Lancet 2024; 404; 803–822. 39121882 10.1016/S0140-6736(24)01029-8

[84] Lei T , Shen M , Deng X *et al*. Genomic characteristics of two breast malignant phyllodes tumors during pregnancy and lactation identified through whole‐exome sequencing. Orphanet J. Rare Dis. 2022; 17; 382. 36271373 10.1186/s13023-022-02537-w PMC9587670

[85] Jardim DL , Conley A , Subbiah V . Comprehensive characterization of malignant phyllodes tumor by whole genomic and proteomic analysis: biological implications for targeted therapy opportunities. Orphanet J. Rare Dis. 2013; 8; 112. 23895135 10.1186/1750-1172-8-112 PMC3751902

[86] Sha H , Liu Q , Xie L *et al*. Case report: pathological complete response in a lung metastasis of phyllodes tumor patient following treatment containing peptide neoantigen Nano‐vaccine. Front. Oncol. 2022; 12; 800484. 35211402 10.3389/fonc.2022.800484 PMC8861377

[87] Suzuki S , Saito Y . Genomic analysis of advanced phyllodes tumors using next‐generation sequencing and their chemotherapy response: a retrospective study using the C‐CAT database. Medicina (Kaunas) 2024; 60; 1898. 39597083 10.3390/medicina60111898 PMC11596819

[88] Conduit C , Luen S , Xu H *et al*. Using genomic sequencing to explain an exceptional response to therapy in a malignant phyllodes tumor. JCO Precis. Oncol. 2020; 4; 1263–1266. 35050785 10.1200/PO.20.00305

[89] Chu X , Wu M , Yang J *et al*. Organoid models derived from patients with malignant phyllodes tumor of the breast. Breast Cancer Res. Treat. 2023; 200; 193–201. 37204665 10.1007/s10549-023-06973-5

